# Intratracheal Administration of Mesenchymal Stem Cells Modulates Tachykinin System, Suppresses Airway Remodeling and Reduces Airway Hyperresponsiveness in an Animal Model

**DOI:** 10.1371/journal.pone.0158746

**Published:** 2016-07-19

**Authors:** Konrad Urbanek, Antonella De Angelis, Giuseppe Spaziano, Elena Piegari, Maria Matteis, Donato Cappetta, Grazia Esposito, Rosa Russo, Gioia Tartaglione, Raffaele De Palma, Francesco Rossi, Bruno D’Agostino

**Affiliations:** 1 Department of Experimental Medicine, Section of Pharmacology, Second University of Naples, Naples, Italy; 2 Department of Clinical and Experimental Medicine, Second University of Naples, Naples, Italy; Cincinnati Children's Hospital Medical Center, UNITED STATES

## Abstract

**Background:**

The need for new options for chronic lung diseases promotes the research on stem cells for lung repair. Bone marrow-derived mesenchymal stem cells (MSCs) can modulate lung inflammation, but the data on cellular processes involved in early airway remodeling and the potential involvement of neuropeptides are scarce.

**Objectives:**

To elucidate the mechanisms by which local administration of MSCs interferes with pathophysiological features of airway hyperresponsiveness in an animal model.

**Methods:**

GFP-tagged mouse MSCs were intratracheally delivered in the ovalbumin mouse model with subsequent functional tests, the analysis of cytokine levels, neuropeptide expression and histological evaluation of MSCs fate and airway pathology. Additionally, MSCs were exposed to pro-inflammatory factors *in vitro*.

**Results:**

Functional improvement was observed after MSC administration. Although MSCs did not adopt lung cell phenotypes, cell therapy positively affected airway remodeling reducing the hyperplastic phase of the gain in bronchial smooth muscle mass, decreasing the proliferation of epithelium in which mucus metaplasia was also lowered. Decrease of interleukin-4, interleukin-5, interleukin-13 and increase of interleukin-10 in bronchoalveolar lavage was also observed. Exposed to pro-inflammatory cytokines, MSCs upregulated indoleamine 2,3-dioxygenase. Moreover, asthma-related *in vivo* upregulation of pro-inflammatory neurokinin 1 and neurokinin 2 receptors was counteracted by MSCs that also determined a partial restoration of VIP, a neuropeptide with anti-inflammatory properties.

**Conclusion:**

Intratracheally administered MSCs positively modulate airway remodeling, reduce inflammation and improve function, demonstrating their ability to promote tissue homeostasis in the course of experimental allergic asthma. Because of a limited tissue retention, the functional impact of MSCs may be attributed to their immunomodulatory response combined with the interference of neuropeptide system activation and tissue remodeling.

## Introduction

Asthma affects hundreds of millions of people and its growing incidence calls for more research [1]. In asthma, inflammation and epithelial damage favor remodeling of the airway wall and airway hyperresponsiveness (AHR). These dynamic phenomena involve a thickening of the airway epithelium, increased number of mucous cells and smooth muscle cell (SMC) hypertrophy and hyperplasia [2,3]. The progressive pathological features correlate with the clinical symptoms, such as airway obstruction, dyspnea and wheezing as well as disease exacerbations. Unfortunately, the therapeutic response varies markedly between individuals, with about 10% of patients showing evidence of drug insensitivity [4]. Therefore, there is a need for new and more effective treatments for refractory asthma in which the clinical manifestations have not been reduced or removed by standard therapy.

Stem cell-based interventions have been recognized as an important issue and continuing progresses have been made in investigating the role of different classes of regionally distinct lung-resident stem/progenitor cells [5–11]. Moreover, extrapulmonary cells including marrow-, adipose tissue- and umbilical cord blood-derived stromal cells, embryonic stem cells and induced pluripotent stem cells were tested in pulmonary settings [12,13]. Mesenchymal stem cells (MSCs) are adult stem cells traditionally found in the bone marrow, but they have also been identified and isolated from other tissues including the lung [14]. In addition to their well-known ability to acquire connective tissue lineages, such us fat, cartilage and bone [15], several *in vitro* studies have demonstrated that MSCs can also differentiate into cells of non-mesenchymal origin (i.e. bronchial epithelium, neuronal tissue and cardiomyocytes) [16,17]. Nonetheless, because of still uncertain MSC plasticity *in vivo*, current evidence indicates that MSC-dependent functional improvements of target organs are to be accredited more to an indirect participation to tissue repair than to their widespread engraftment and transdifferentiation [18,19]. Additionally, MSCs exhibit strong immunomodulatory potential via the interaction with T lymphocytes, B lymphocytes, natural killer cells and dendritic cells [20–23]. At the same time, low expression of HLA class I and the lack of MHC II and co-stimulatory molecules make MSCs reasonable candidates for allogeneic transplantation. The secretion of numerous growth factors and the expression of surface molecules make these cells capable of modulating the function of host cells within the injured environment, both by cell-to-cell contact and paracrine mechanisms [24,25]. Preclinical studies have reported promising results for the efficacy of MSC therapy in numerous lung disorders, including emphysema [26,27], acute lung injury [28,29], bronchopulmonary dysplasia [30], pulmonary arterial hypertension [31], lung fibrosis [32,33], obliterative bronchiolitis [34] and asthma [35–38], and these robust evidence have provided the basis for clinical trials [12,39]. Despite that, mechanisms by which MSCs exert their action in lung diseases are understood only in part. Moreover, the data regarding effects of locally administered MSCs are scarse. Therefore, the aim of our study was to investigate the role of MSCs in interfering with pathophysiological features of airway hyperresponsiveness, with a particular interest in the impact of MSCs on airway remodeling and local neuropeptide systems after local administration.

## Materials and Methods

### MSC isolation and culture

Mouse MSCs were isolated from bone marrow of 6 weeks-old BALB/c mice as previously described [40]. Femurs and tibias were dissected from attached muscle and connective tissue and washed several times with PBS. The ends of the bones were removed, and marrow was extruded by inserting a needle into the bone shaft and flushing it with α-MEM supplemented with 10% FBS, penicillin (100 U/ml), streptomicin (100 mg/ml). The cells were washed twice with PBS and seeded at a density of 7x10^4^ cells/cm^2^. The non-adherent cell population was removed after 48 h, the adherent layer washed once with PBS and fresh medium was added. The cells were used from passage 1 to 3.

### FACS analysis

FACS analysis was performed for MSC phenotype characterization. In particular, PE-conjugated antibodies for CD105, CD90, CD73, CD44, CD45 and CD31 were used (BD Biosciences, Italy). Isotype-matched negative control was utilized to define the threshold for each specific signal. Cells were analyzed by FACS (FACScalibur, BD Biosciences).

### Stimulation of MSCs with inflammatory cytokines

MSCs (1.5 x10^5^ cells) were seeded in 60 mm diameter culture dishes in regular culture medium and were simultaneously stimulated with TNFα (10 ng/ml) and IFNγ (10 ng/ml) to mimic inflammatory environment [41]. Total RNA was extracted after 3, 6, 12 and 24 h.

### RNA extraction from cells and Quantitative RT-PCR

Total RNA was extracted with TRIzol from untreated and stimulated MSCs for the detection of transcripts for IDO, TGF-β and IL-10 (KiCqStart SYBR Green Primers; Sigma Aldrich, Germany). HPRT was used as housekeeping gene (KiCqStart SYBR Green Primers; Sigma Aldrich). iScript One-Step RT-PCR Kit with SYBR Green (Bio-Rad Laboratories, Italy) was employed to perform Real-time PCR and 3 ng of total mRNA from each sample was used as template. Cycling conditions were set according to manufacturer’s instructions: cDNA synthesis (10 min at 50°C); reverse transcriptase inactivation (5 min at 95°C); PCR cycling and detection (42 cycles; 10 sec at 95°C; 30 sec at 58°C); melt curve analysis (1 min at 95°C, 1 min at 55°C, 5 sec at 55–95°C, increasing by 0.5°C each cycle). A CFX96 Real-time PCR Detection System was employed (Bio-Rad Laboratories).

### Lentiviral transduction

After expansion, 8x10^5^–1x10^6^ MSCs were transduced with a Cignal Lentivirus carrying GFP and puromycin resistance genes at a MOI of 50. After 18–20 h, cells were washed and infection medium was replaced by fresh medium. At this time, Cignal reporter constructs are integrated into the genomic DNA. To select the cells stably expressing the reporter GFP gene, puromycin (5 μg/ml) selection was performed for additional two weeks and cells were detached, collected by centrifugation, diluted at the density of 5x10^4^ cells/50 μl in the appropriate medium and used for *in vivo* studies.

### *In vivo* experimental protocol

**Fig 1:**
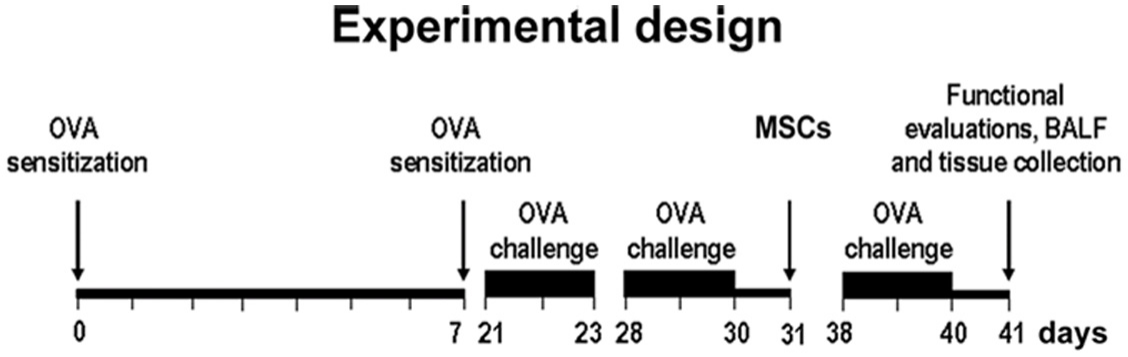
Experimental Design. Scheme of *in vivo* experiments.

To induce AHR, BALB/c mice at 6 weeks of age were sensitized by two s.c. injections of 0.4 ml of 10 μg OVA, absorbed to 3.3 mg of aluminum hydroxide gel in sterile saline at days 0 and 7. From day 21, mice were challenged by inhalation with nebulized OVA (1% in PBS) for 7 min, three days per week for three weeks by an ultrasonic nebulizer (De Vilbiss Health Care, UK). OVA derived from chicken egg is a frequently used allergen that induces an allergic pulmonary inflammation in laboratory rodents [42,43]. Mice were randomized into three experimental groups: 1. Control (n = 12), not subjected to any treatment, received s.c. injections of saline followed by saline inhalations; 2. OVA (n = 18), sensitized and challenged with OVA and injected with medium; 3. OVA+MSCs (n = 18), sensitized and challenged with OVA and treated with MSCs. Medium or MSCs were intratracheally administered on day 31, 24 h after the second week of OVA challenge. All mice were sacrificed 10 days after intratracheal administration of MSCs or medium and lung reactivity test or BAL were performed. Separate sets of animals were used for lung reactivity assay or BAL collection because of the possibility that manipulations of the lungs during BAL procedure affect lung reactivity measurements. After the assessment of lung reactivity, lungs were perfused and fixed with 10% phosphate-buffered formalin for histology. A schematic representation of the study protocol is shown in Fig 1. Six control animals were treated with MSCs to verify cell engraftment and potential functional impact on the healthy lung.

### Intratracheal administration of MSCs

Prior to cell administration, mice were anesthetized with ketamine HCl 40 mg/kg i.p. and medetomidine hydrochloride 0.15 mg/kg i.p. A 20-gauge custom-made catheter was inserted into the trachea via the mouth, and connected to a mouse ventilator (Harvard Apparatus, MA, USA). After confirming the correct position of the catheter in the trachea and disconnecting the ventilator, 5x10^4^ cells/50 μl medium were delivered into OVA+MSCs animals through the catheter. Afterwards, mice were mechanically ventilated for 3 min, and placed in a warm chamber until they recovered consciousness, usually within 5–15 min. Mice from the OVA group received equal volume of medium.

### Lung reactivity assay

Lung reactivity was assessed by isolated and perfused mouse lung technique. As previously described [44], water-jacketed (water temperature, 37°C) acrylic glass chamber was used to accommodate surgery, perfusion and ventilation. Mice were anesthetized with ketamine HCl 40 mg/kg i.p. and medetomidine hydrochloride 0.15 mg/kg i.p. The trachea was exposed and cannulated after a small incision to allow the connection to the pneumotachograph. The diaphragm was cut and 50 μl of heparin were injected into the heart. In order to obtain an optimal perfusion of pulmonary artery, anesthetized mice were exsanguinated by the incision of the renal vein, the thorax was opened and the two thoracic halves were immobilized with two pins at sides on the cork plate. At this point pulmonary artery was cannulated through the right ventricle, so that the arterial cannula was inserted into the pulmonary artery and fixed by the ligature. The lungs were perfused through the pulmonary artery in a non-recirculating fashion at a constant flow of 1 ml min^−1^ resulting in a pulmonary artery pressure of 2–3 cm H_2_O. As a perfusion medium, RPMI 1640 lacking phenol red (37°C) enriched with 4% low endotoxin grade albumin was used. The lungs were ventilated by external negative pressure (−3 to −9 cm H_2_O) with 90 breaths min^−1^ and a tidal volume of about 200 μl. Every 5 min a hyperinflation (−20 cm H_2_O) was performed. Artificial thorax chamber pressure was measured with a differential pressure transducer (Validyne DP 45–24, Validyne Engeneering, CA, USA), and airflow velocity with a pneumotachograph tube connected to a differential pressure transducer. The lungs respired humidified air. The arterial pressure was continuously monitored with a pressure transducer (Isotec; Healthdyne Cardiovascular, CA, USA) connected with the cannula ending in the pulmonary artery. All data were transmitted to a computer and analyzed by the Pulmodyn software (Hugo Sachs Elektronik, Germany). For lung mechanics, the data were analyzed by applying the following formula: P = V•C−1 + RL•dV•dt−1, where P is chamber pressure, C pulmonary compliance, V tidal volume and RL airway resistance. After 60 min, mean tidal volume was 0.21±0.02 ml, mean airway resistance 0.23±0.08 cm H_2_O s ml^−1^, and mean pulmonary artery pressure 2.9±1.4 cm H_2_O. The measured airway resistance was corrected for the resistance of the pneumotachometer and the tracheal cannula of 0.6 cm H_2_O s ml^−1^. Increasing concentrations of acetylcholine (ACh; 10^−8^ M to 10^−3^ M) were administered in 5 min intervals through the pulmonary artery cannula and a dose response curves were obtained in all experimental groups. Each dose of ACh was separated by a buffer washout.

### Bronchoalveolar lavage

BAL was performed as follows: 1.5 ml of saline was instilled and withdrawn from the lungs via an intratracheal cannula; this lavage was performed three times, and different samples were collected. The BAL fluid was centrifuged at 1000 g for 10 min at 4°C. The supernatant was transferred into tubes and stored at −70°C for analysis of cytokines. Cell pellets were resuspended in PBS to a final volume of 500 μl for total and differential cell count.

### Total and differential cell count

Total cell count was performed with the Countess automated cell counter (Life Technologies, Italy) which evaluates cell number and viability using trypan blue stain according to the manufacturer’s instructions. Differential counting was performed on Reastain Diff-Quik stained cytospins and at least 300 cells were counted on each preparation according to standard morphologic criteria under light microscopy.

### Cytokines assay

Measurement of cytokines in the BAL were performed taking advantage of a well-established method, Luminex xMAP technology (Luminex^®^ 200^™^ System, Life technologies), that allows to measure a panel of multiple analytes on a small volume sample (100 μl) simultaneously [45]. The assays, for the quantitative detection of IL-4, IL-5, IL-10 and IL-13, were performed using a Milliplex Cytokine Panel plate (Millipore-Merck, Italy) according to the manufacturer’s instructions on automated immunoassay analyzer as previously described [46]. All samples were run in duplicate. After the run, data were analyzed using by Xponent software (1.9 version, Luminex^®^ 200^™^ System, Life technologies) and the final concentration of each cytokine expressed in pg/ml.

### Histochemistry and immunofluorescence

Lungs were perfused and fixed with 10% phosphate-buffered formalin for 15 min. Perfusion pressure was kept at 2–3 cm H_2_O. Subsequently, the lungs were excised, immersed in formalin for 24 h, and embedded in paraffin. Tissue sections, 5 μm in thickness, were used for histological analysis. Injected cells were detected by anti-GFP antibody (abcam, UK); lung cells were identified by immunostaining for CFTR, TTF1, pan-CK (abcam) and surfactant protein-C (SPC) (Santa Cruz Biotechnology, CA, USA); SMCs were detected with anti-SMA (Sigma-Aldrich); inflammatory cells were detected with CD45 and CD3 antibodies (Novus Biologicals, CO, USA). Cycling cells were visualized using anti-Ki67 antibody (Vector Laboratories, UK) Nuclei were stained with DAPI (Sigma-Aldrich). Secondary antibodies conjugated with FITC or TRITC were used (Jackson ImmunoResearch, UK). At the end, sections were stained with Sudan black. Four sections per animal were stained and five to ten images per section were used for airway remodeling quantification.

For the assessment of inflammation, sections were stained with H&E. The number of mast cells per mm^2^ of the lung tissue, was measured after staining with toluidine blue (Sigma-Aldrich). Five tissue sections per animal were stained and the whole area was examined. Tissue sections were stained with Masson’s thricrome staining (Sigma-Aldrich) for visualization of structural elements. The cross-sectional area of airway smooth muscle mass and internal perimeter of the basement membrane were measured in bronchial profiles. The square root of area of airway smooth muscle mass was then corrected by the perimeter of the basement membrane [47]. Five animals form each experimental group were used for airway smooth muscle mass measurements. Three tissue sections per animal were stained and seven to eleven bronchi per section were used for quantification. Morphologic measurements were done with Image Pro Plus software (Media Cybernetics, MD, USA). Mucicarmine (Mucin Stain) kit was used for the visualization of acid mucopolysaccharides in tissue sections according to manufacturer’s instructions (abcam). Additionally, the number of mucous producing cells was assessed by the immunolabelling with anti-mucin 5AC antibody (abcam). Mucin-positive cells were quantified in the epithelial layer of the bronchi by counting labeled cells per total number of cells within the airway epithelium. Samples were analyzed with a Leica DM 5000B microscope a Zeiss LSM 700 confocal microscope.

### PCR for detection of GFP DNA in the tissue

For PCR detection of GFP, paraffin sections were obtained from the lungs of mice in which GFP-positive cells were previously detected by immunohistochemistry. Tissue sections were deparaffinized and genomic DNA was extracted with the QIAamp DNA kit (Qiagen, Italy). DNA, 100 ng, was mixed with primers for GFP (GFP-F: 5'-ATGGTGAGCAAGGGCGAGGAGCTG-3' and GFP-R: 5'-GCCGT-CGTCCTTGAAGAAGATGGTG-3'). Cycling conditions were as follows: 94°C for 30 sec, followed by 30 cycles of amplification (94°C for 30 sec, 62°C for 30 sec, 72°C for 30 sec), with a final incubation at 72°C for 3 min. PCR products were run onto agarose gel for the detection of the GFP band (amplicon size: 315 bp). DNA extracted from tissue sections of mice injected with medium were used as negative controls [48].

### RNA extraction from tissue and Quantitative RT-PCR

Total RNA were extracted with TRIzol from lungs obtained from each experimental group for the detection of transcripts for calcitonin gene-related peptide (CGRP), vasoactive intestinal peptide (VIP), neurokinin 1 receptor (NK1-R) and neurokinin 2 receptor (NK2-R) (KiCqStart SYBR Green Primers; Sigma Aldrich). HPRT was used as housekeeping gene. iScript One-Step RT-PCR Kit with SYBR Green was employed to perform RT-PCR and 60 ng of total mRNA from each sample was used as template. Cycling conditions were performed according to manufacturer’s instructions: cDNA synthesis (10 min at 50°C); reverse transcriptase inactivation (5 min at 95°C); PCR cycling and detection (42 cycles; 10 sec at 95°C; 30 sec at 58°C); melt curve analysis (1 min at 95°C, 1 min at 55°C, 5 sec at 55–95°C, increasing by 0.5°C each cycle). A CFX96 RT-PCR Detection System was employed. Quantified values were normalized against the input determined by the housekeeping gene.

### Statistical Analysis

Results are reported as mean ± SD or SEM. Significance for multiple comparisons was determined by one-way ANOVA and Bonferroni’s post-test. Lung reactivity curves were compared using a two-way ANOVA followed by Bonferroni post-test. A value of P<0.05 was considered as significant; and the actual P values were included in the figures. To avoid inter-operator variability, a single operator blinded to the animal groups conducted every data analysis.

### Animal studies approval

The investigation was approved by the Veterinary Animal Care and Use Committee of the Second University of Naples (permit n. 1961/2012) and conforms to the National Ethical Guidelines of the Italian Ministry of Health and the Guide for the Care and Use of Laboratory Animals (National Institute of Health, Bethesda, MD, USA, revised 1996). BALB/c mice at 5 weeks of age were obtained from Harlan Laboratory (Udine, Italy).

### Animal housing

Mice were housed in the animal facility of the Second University of Naples. Food and water were supplied *ad libitum*. Room temperature was 22°C–24°C, relative humidity was 40%–50%, and the day/night cycle was set at 12 h/12 h. Mice were acclimatized for 1 week before starting any procedures and during this time, they were submitted to a daily handling to get them used to manipulation, thus reducing experimental variability due to the stress given by procedures. All treatments were performed by experienced operators and in asepsis. During challenge (aerosol), freely moving mice were kept in a suitable chamber with the minimal stress. Prior to the experimental endpoint, no animal became severely ill or died at any time. In order to prevent and exclude any possible animal pain, all experimental animals were anesthetized with ketamine and medetomidine hydrochloride. The mice subjected to lung reactivity test were exsanguinated after incision of the renal vein as required by the assay procedure (see above), while the remaining animals were sacrificed by cervical dislocation.

## Results

### Airway responsiveness

**Fig 2:**
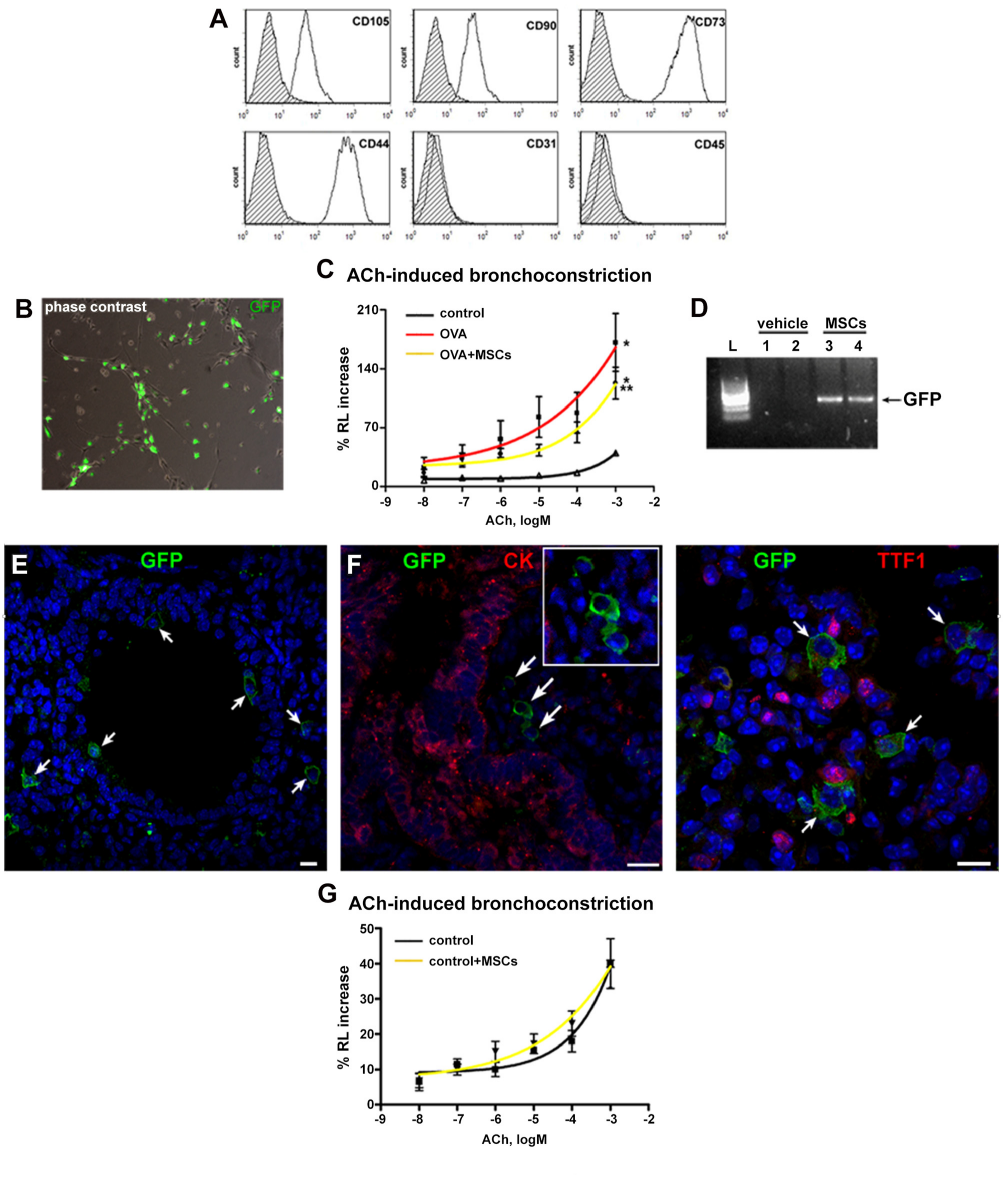
MSCs are retained within the lungs and improve function *in vivo*. (**A**) FACS analysis of MSCs for mesenchymal, hematopoietic and endothelial markers. (**B**) Phase contrast and GFP fluorescence image of living MSCs after lentiviral transduction. (**C**) Airway reactivity to ACh as change in resistance (RL) in control, asthmatic (OVA) and MSC-treated (OVA+MSCs) asthmatic mice. (**D**) Detection of GFP gene by PCR in the lungs of MSC-treated mice. (**E**) GFP-positive cells (green, arrows) in MSCs-treated mice. (**F**) GFP-positive MSCs (green, arrows and inset) lack the expression of epithelial markers CK and TTF1 (red). (**G**) Airway reactivity to acetylcholine as change in resistance in control mice and control mice after instillation of MSCs. Scale bars 20 μm. *P<0.05 vs control; **P<0.05 vs OVA. Four to eight mice were used for airway function assessment. OVA: ovalbumin; ACh: acetylcholine; CK: pan-cytokeratin.

Prior to use for *in vivo* experiments, MSCs were characterized by FACS. Specifically, undifferentiated MSCs typically expressed CD105, CD90, CD73 and CD44, while failed to express hematopoietic and endothelial markers, such as CD45 and CD31 (Fig 2A). Afterwards, to determine whether MSCs can interfere with the airway responsiveness, GFP-tagged MSCs (Fig 2B) were intratracheally instilled in animals sensitized and challenged with OVA. OVA mice had increased ACh-induced bronchoconstriction. With respect to OVA mice, in the animals that received MSCs, a significant reduction in bronchial hyperreactivity was observed (Fig 2C). These results indicate that MSCs, after local administration, are able to partially restore normal bronchial reactivity.

### Engraftment and *in vivo* differentiation of MSCs

The critical question for cell therapy is whether administered cells engraft to the target tissue. Ten days after cell administration, PCR and immunohistochemistry revealed the presence of GFP gene and GFP-labeled cells in the lungs of MSC-treated animals (Fig 2D and 2E). In MSC-treated animals, only scattered GFP-positive cells were present (Fig 2E). MSCs did not acquire lung phenotype *in vivo*, as tested by double staining for GFP and lung markers CK, TTF1 (Fig 2F), CFTR and SPC (not shown). To determine the fate of MSCs in the absence of inflammation, cells were given to control mice. In these animals, the injected cells were not detected, indicating that tissue damage creates the permissive environment for MSC engraftment. Moreover, in a normal lung, injected cells did not affect airway reactivity (Fig 2G).

### Airway remodeling: epithelial cell, smooth muscle cell and goblet cell hyperplasia

**Fig 3:**
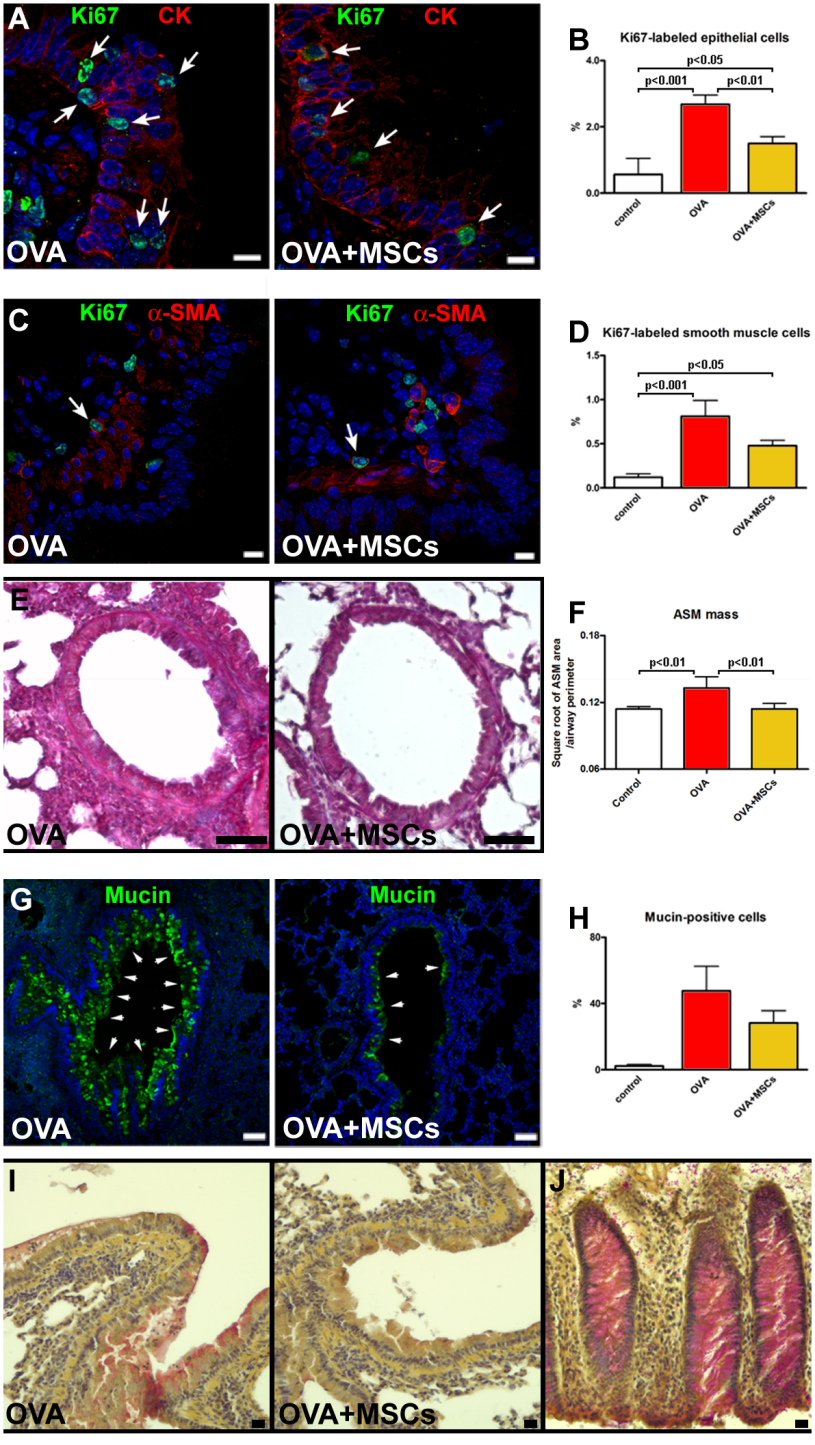
MSCs positively interfere with airway remodeling. (**A**) Proliferating (Ki67, green, arrows) epithelial cells (CK, red) in vehicle-treated and MSC-treated animals. (**B**) The fraction of Ki67-positive epithelial cells. (**C**) Cycling (Ki67, green, arrows) SMCs (α-SMA, red) in the airway wall. (**D**) The fraction of Ki67-positive SMCs. (**E**) Airway smooth muscle mass in OVA and OVA+MSC mice. (**F**) Quantification of airway smooth muscle mass. (**G**) Mucin-positive cells (green) in epithelium of vehicle-treated and MSC-treated asthmatic mice. (**H**) The percentage of epithelial cells expressing mucin. (**I**) Acid mucopolysaccharides accumulation in OVA and OVA+MSCs groups. (**J**) Human colon as positive control for mucicarmine staining. Scale bars (A and C) 10 μm, (E and G) 50 μm, (I and J) 20 μm. Five to seven animals were used for airway remodeling data. CK: pan-cytokeratin; α-SMA: α-smooth muscle actin.

Since epithelial cell hyperplasia has been documented in asthmatic subjects [49], the expression of cell cycle marker in epithelial cells was measured. In OVA mice, the fraction of Ki67-positive cells was significantly higher than in controls. However, the fraction of cycling epithelial cells was reduced in MSC-treated mice (Fig 3A and 3B). Cycling SMCs were detected in the airways of all experimental groups. While in the OVA group the fraction of Ki67-positive SMCs was significantly higher than in controls, in cell-treated mice this parameter was markedly lower (Fig 3C and 3D). This finding together with the measurement of smooth muscle layer suggested that cell therapy attenuated the increase of smooth muscle mass observed n OVA mice (Fig 3E and 3F). Finally, an increase in mucus-producing cells observed in OVA mice, was moderately lowered by MSCs as detected by mucin immunolabeling (Fig 3G and 3H) and mucicarmine staining (Fig 3I and 3J).

### Inflammation and immunomodulation

**Fig 4:**
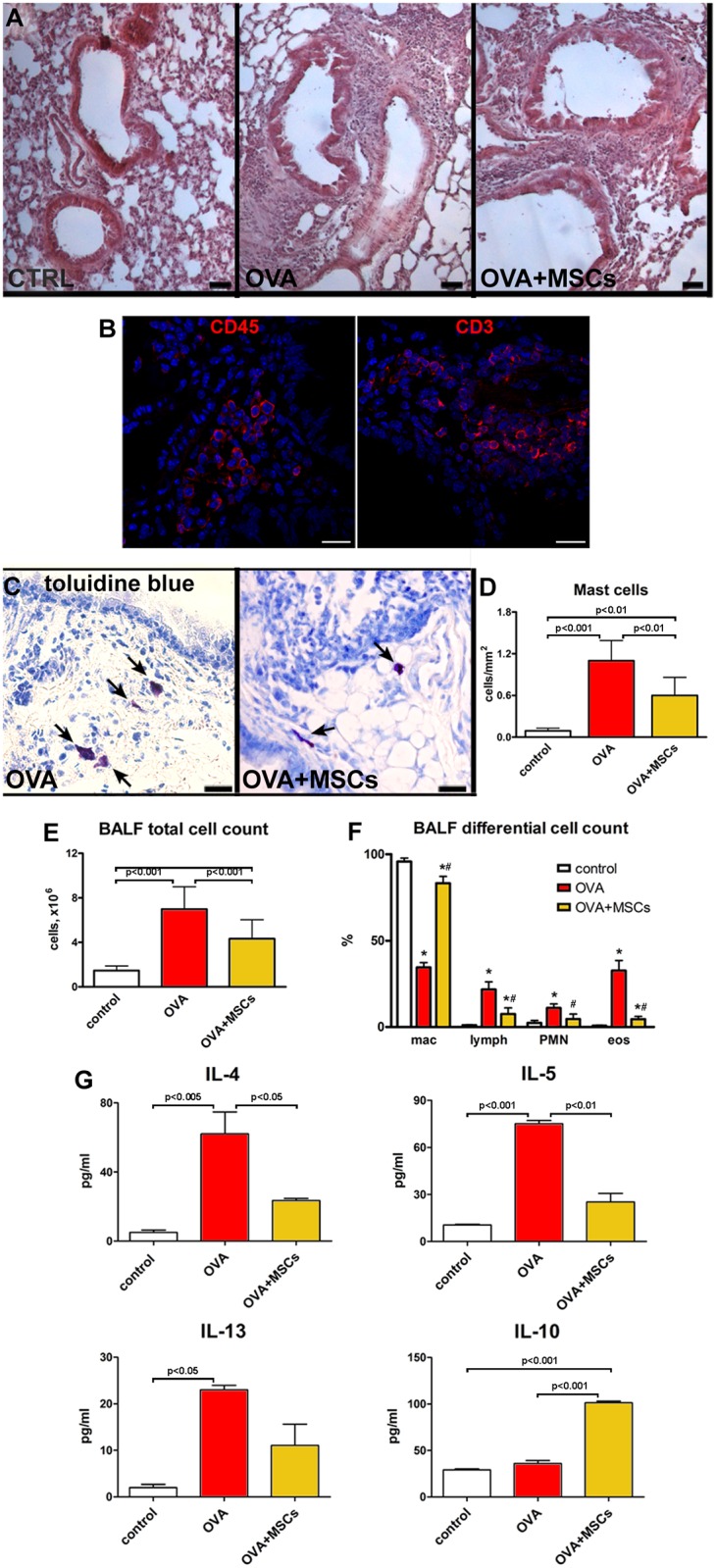
MSCs decrease lung inflammation. (**A**) With respect to controls, massive accumulation of inflammatory cells visible in the lungs of OVA animals was reduced in MSC-treated asthmatic mice. (**B**) Detection of CD45 and CD3 positive cells (red) within the inflammatory milieu. (**C**) Mast cells (toluidine blue, arrows) in the lungs of vehicle- and MSC-treated asthmatic mice. (**D**) The number of mast cells per mm^2^ of tissue. (**E,F**) Total cell number and differential cell count in the BALF collected from control, OVA and OVA+MSCs animals. (**G**) Cytokines levels measured in the BALF. Scale bars (A) 50 μm, (C) 20 μm. *P<0.05 vs control; #*P*<0.05 vs OVA. Four to ten animals were used for mast cell count. BALF from three to ten mice were used for cell count and cytokine assay. BALF: bronchoalveolar lavage fluid; mac: macrophages; lymph: lymphocytes; PMN: polymorphonuclear leukocytes; eos: eosinophiles.

Histological analysis revealed a massive peri-bronchial accumulation of inflammatory cells in OVA mice. After MSC administration, the extent of inflammatory infiltration was markedly lowered (Fig 4A). The expression of CD45 and CD3 markers in the cells within infiltration surrounding the bronchi confirmed their inflammatory phenotype (Fig 4B). Additionally, the increased number of mast cells observed in OVA animals was significantly reduced after cell treatment (Fig 4C and 4D). The analysis of BAL showed an increase in a total cell number in OVA mice, with typical changes in differential cell count. BAL from cell-treated animals showed the reduction in total cell number. When compared with OVA group, MSCs significantly reduced the proportion of eosinophils and lymphocytes and increased the fraction of macrophages (Fig 4E and 4F). Such an increase, already reported for MSCs, can suggest macrophage activation to promote anti-inflammatory function and tissue repair [50].

BAL from OVA mice had significantly increased concentrations of Th2 pro-inflammatory cytokines such as IL-4, IL-5 and IL-13. Cell treatment reduced the levels of Th2 pro-inflammatory cytokines and increased the level of IL-10 (Fig 4G). Taken together, MSCs had a positive modulatory effect not only on the airway remodeling but also on the inflammatory process, confirming their immunomodulatory properties.

**Fig 5:**
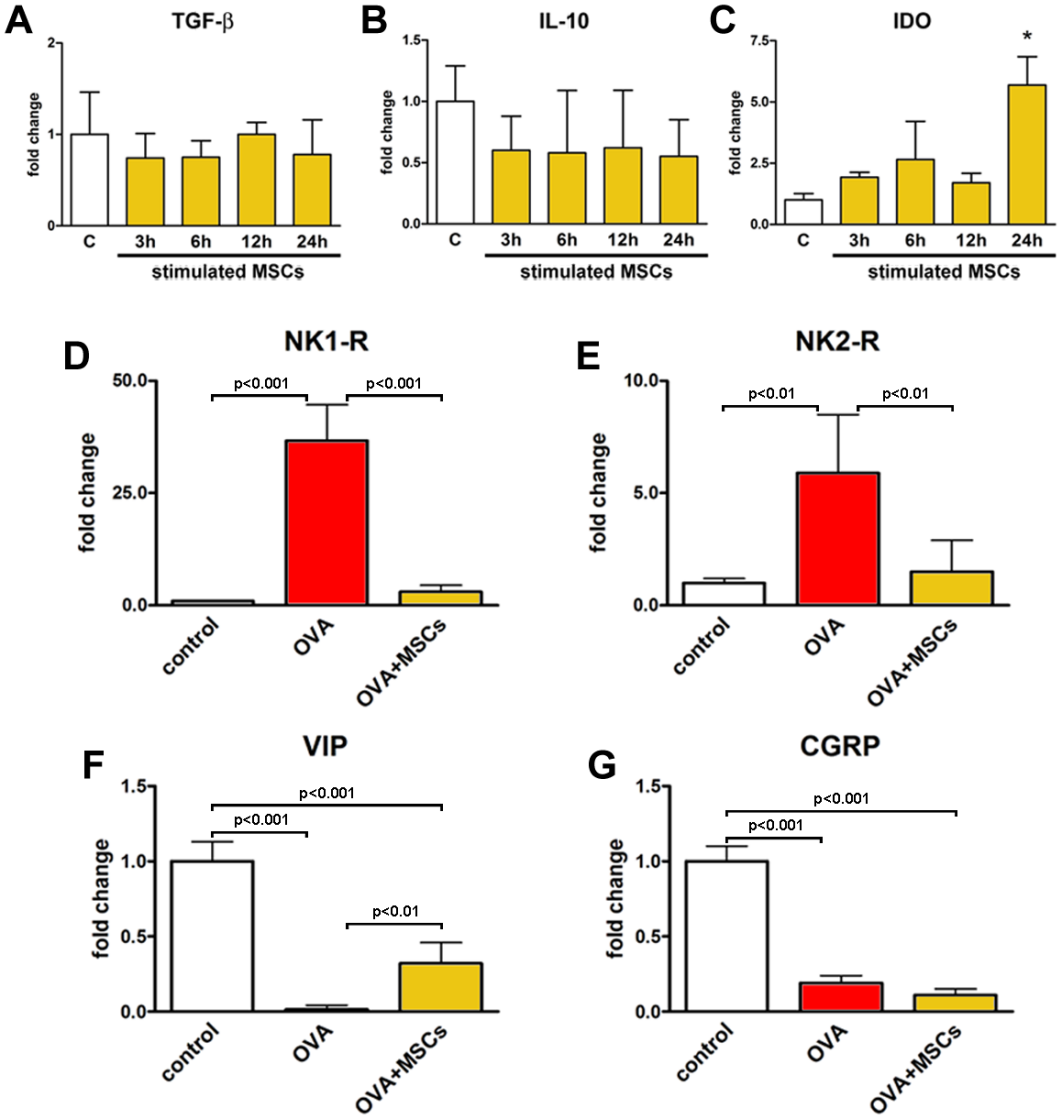
Immunomodulatory properties of MSCs. (**A-C**) Expression of mRNA for TGF-β, IL-10 and IDO measured by real-time RT-PCR in control (C, white bars) and MSCs (yellow bars) stimulated with IFNγ and TNFα after 3, 6, 12 and 24 h. *P<0.05 vs control cells. Expression of mRNA for (**D-G**) NK1-R, NK2-R, VIP and CGRP measured by real-time RT-PCR in control, OVA and OVA+MSCs groups. Three sets of MSCs were used for *in vitro* experiments. Four to seven animals were used for PCR analysis of neuropepetides. TGF-β: transforming growth factor-β; IDO: indoleamine 2,3-dioxygenase; CGRP: calcitonin gene-related peptide; VIP: vasoactive intestinal peptide; NK1-R; neurokinin 1 receptor; NK2-R: neurokinin 2 receptor.

In the search for the potential mechanisms responsible for the positive effects of MSCs on the inflammation, isolated cells were stimulated with IFNγ and TNFα, and the expression levels of mRNA for TGF-βIL-10 and IDO were measured. Real time PCR showed the presence of TGF-β and IL-10 transcripts in MSCs, but no difference in expression was revealed after pro-inflammatory stimulation (Fig 5A and 5B). On the contrary, stimulated MSCs had increased levels of IDO mRNA as compared with non-stimulated cells (Fig 5C). A large body of evidence points to the neurogenic inflammation and peptidergic neuromediators in airway inflammatory diseases [51]. Of note, the expression of pro-inflammatory NK1-R and NK2-R receptors, that are principally activated by substance P and neurokinin A, was increased in the lungs of OVA group and significantly reduced by MSC treatment (Fig 5D and 5E). Additionally, the level of anti-inflammatory neuropeptide VIP, almost completely blunted in asthmatic mice, was partially restored by MSC therapy (Fig 5F). Conversely, CGRP expression, that was decreased in OVA mice, resulted unchanged by MSCs (Fig 5G). These data indicate that MSCs, by promoting the downregulation of NK1-R and NK2-R, along with the upregulation of VIP, may affect the activation of pulmonary peptidergic signaling in pathological conditions.

## Discussion

Asthma consists of intermittent airway obstruction, bronchial hyperreactivity and chronic inflammation with airways' structural changes. The optimal treatment strategy should relieve symptoms, attenuate inflammation and prevent remodeling [2]. The need for new options for patients suffering from chronic lung diseases has boosted the research on stem cells for lung repair [5]. However, due to the complexity of the endogenous lung stem/progenitor cells, the interest in testing stem cells from extrapulmonary sources has also been growing. While functional benefits of MSCs and their immunomodulatory properties have been reported, the data on cellular processes involved in early airway remodeling and the potential involvement of neuropeptides are largely missing. The immunomodulatory plasticity of MSCs is an intriguing issue. An emerging body of evidence has clarified that MSCs, in the presence of an inflammatory environment (high levels of TNFα and IFNγ), adopt an immune-suppressive phenotype by secreting high levels of soluble factors, including IDO and prostaglandin E2, which suppress T cell proliferation. Otherwise, in the absence of an inflammatory environment (low levels of TNFα and IFNγ), MSCs may adopt a pro-inflammatory phenotype and enhance T cell responses by secreting chemokines that recruit lymphocytes to the site of inflammation [52]. In our model of OVA-induced AHR, MSCs were delivered after OVA sensitization and the second challenge with OVA aerosol when lung tissue is already characterized by an inflammatory milieu in which inflammation processes are active. The data presented here confirm some of known effects and extend the existing knowledge regarding the mechanisms by which MSCs can modulate airway inflammatory milieu and interfere with progressive structural changes. Of note, while the vast majority of the published work has utilized the systemic infusion of cells [36–38,50,53,54], local administration was used only in isolated reports [55,56]. The intratracheal route for cell delivery has particular advantages. It allows reducing number of cells, puts them in the place of need without the necessity to traverse vessel wall and minimizes the risk of colonization of other organs.

In this study, after intratracheal administration, MSCs partially restored normal bronchial reactivity. At the same time, a direct conversion into the lung lineages was not detected pointing to the possibility that functional benefits were due to an indirect mode of action. This is in line with the findings demonstrating that MSCs are able to mitigate AHR despite of the low retention within the host tissue [28,55,57].

Progression of asthma is characterized by hypertrophy and hyperplasia within the bronchial smooth muscle layer accompanied by epithelial hyperplasia and increase in number of goblet cells [2,38]. In our study, MSC-treated animals had lower cycling index of airway SMCs and airway smooth muscle mass, indicating that cell therapy reduced the hyperplastic phase of smooth muscle mass increase. Additionally, treatment with MSCs decreased the proliferation rate of pre-existing epithelium that together with the reduction in the excessive production of airway mucus glycoproteins indicate the capacity of locally administered MSCs to interfere with pathological hallmarks of asthma.

In parallel, the beneficial effects of MSCs on inflammatory infiltration of lung tissue and AHR were associated with a decreased number of inflammatory cells and a reduction of pro-inflammatory cytokines IL-4, IL-5 and IL-13 in BAL. At the same time, IL-10, a potent inhibitor of monocyte/macrophage function and eosinophil survival that suppresses the production of a number of pro-inflammatory cytokines [58], was significantly increased. The observed effects are consistent with the current understanding of the primary inflammatory lesion in allergic asthma that consists of the accumulation of CD4 T helper type 2 (Th2) lymphocytes and infiltration of mast cells, basophils, monocytes and eosinophils [59]. Th2 cells orchestrate the inflammation through the secretion of a series of cytokines, particularly IL-4, IL-5 and IL-13, inducing an abnormal shift in the Th1/Th2 balance in favor of Th2 cells. Our data reinforce the notion that MSCs can be effective mostly because of their anti-inflammatory and immunomodulatory properties [12,19,38]. Interestingly, the evidence that resting MSCs can produce IL-10 but do not upregulate this cytokine after exposure to pro-inflammatory factors, together with the increase of IL-10 in BAL, supports the possibility that MSCs might have stimulated other cells to secrete this pleiotropic cytokine. In addition, *in vitro* stimulated MSCs markedly increased mRNA for IDO, known to modulate the immune response by inducing a depletion of tryptophan and the formation of pro-apoptotic metabolites [60]. IFNγ and TNFα are the most common cytokines used as an inflammatory stimulus for *in vitro* experimental protocols, able to induce MSCs to secrete molecules involved in the regulation of tissue homeostasis, including NO, IDO and prostaglandin E2 [61]. Although the “inflammatory profile” of these cytokines does not strictly reflect the inflammatory milieu in the OVA-treated lung tissue receiving MSCs, this experimental approach still provides reliable findings about the MSC response after stimulation. These considerations support the hypothesis that both secretion of soluble mediators and cell-to-cell contact may mediate the immunosuppressive and anti-inflammatory action, favoring a shift from a pro-inflammatory to an anti-inflammatory condition [62].

To further explain functional effects of MSCs, the possible role of neuropeptides was investigated. Although several experimental and clinical evidence have brought the attention to the involvement of sensory neuropeptides in pathophysiological processes in the diseased lung, the question whether the neuroinflammatory system can be modulated by cell therapy remains unanswered [63,64]. Our data document that MSC administration led to a significant decrease in the pulmonary expression of NK1-R and NK2-R mRNA, reverting the dramatic increment detected in the lungs of OVA mice. Moreover, MSC administration determined a significant increase of the protective neuropeptide VIP but did not counteract the decreased expression of CGRP. These observations are noteworthy although the development of novel pharmacological approaches, pointing to tachykinin receptor antagonism as a therapeutic key to counteract bronchoconstriction and inflammation, gave inconclusive results when tested in asthmatic patients [65–67]. Our data may be relevant given an undisputed role of the interaction of tachykinin receptors, NK1-R and NK2-R with their endogenous ligands, substance P and neurokinin A in driving inflammation, bronchoconstriction and airway remodeling [51,68,69]. The activation of NK1-R by substance P is known to potently stimulate epithelial goblet cell secretion, whereas the neurokinin A/NK2-R signaling, remarkably upregulated in the lung of asthmatic subjects and mice [70,71], appears to influence a variety of pathological symptoms and processes in asthma, such as inflammation and AHR [51,72,73]. On the other hand, a protective role of neuropeptitergic system has also been documented. VIP influences many respiratory functions, protects bronchial epithelial cells against damage, produces airway relaxation, inhibits SMC proliferation and reduces inflammation [74]. CGRP regulates inflammation and promotes epithelial repair [75]. Indeed, the current development of VIP-based bronchodilatory drugs that overcome VIP's short half-life indicates that the neuropeptide research remains an option [76].

Overall, downregulation of pro-inflammatory NK1-R and NK2-R, and the concomitant upregulation of VIP observed in our study can be considered an additional mechanism by which MSCs can influence airway pathophysiology. At this stage, the precise meaning of unchanged CGRP remains unknown.

In summary, MSCs positively interfere with multiple features of allergic asthma, confirming their therapeutic potential. The previously unrecognized modulation of neuropeptide system together with immunosuppressive effect and modulation of airway remodeling represent different components that converge to improve airway function.

The final proof providing MSCs’ *in situ* direct effect is a remarkable point that has not been resolved and the functional status of an MSC *in vivo*, within the inflammatory milieu remains to be established. Although it has been shown that MSCs express genes that correlate with their functional properties and numerous and complex mechanisms involved in MSC-mediated immunoregulation, the activity of MSCs *in situ* is usually extrapolated from *in vitro* experiments and measured indirectly, aiming at the detection of the effects at the level of environment, cellular components and overall organ function. The direct insight into MSC behavior has been only recently introduced using the laser capture technique and single cell analysis [77]. The immunosuppressive activity is frequently cited as a unique of MSCs. However, the possibility that other adult stromal cells, in particular fibroblasts from different tissues (3T3 mouse embryonic fibroblasts, dermal fibroblasts, lung fibroblasts), exhibit immunomodulatory properties comparable to that of MSCs, has been raised. These studies have reported quite variable findings, suggesting that MSCs and fibroblasts may share, in part, similar anti-inflammatory mechanisms. Fibroblasts are heterogeneous depending on tissue source, and thus potential anti-inflammatory effects may depend on their origin [12]. Furthermore, fibroblasts are less likely to have the same degree of low immunogenicity as do MSCs and can provoke lung inflammation [78]. In our study, we cannot claim that the immunomodulatory action is a distinctive feature possessed only by MSCs, and therefore, the legitimate question whether, and if so, to which extent, different stromal cells affect lung disease should be addressed in a series of comparison studies.
